# China's COVID-19 Control Strategy and Its Impact on the Global Pandemic

**DOI:** 10.3389/fpubh.2022.857003

**Published:** 2022-03-14

**Authors:** Difeng Ding, Ruilian Zhang

**Affiliations:** ^1^School of Marxism, Hohai University, Nanjing, China; ^2^Sustainable Minerals Institute, The University of Queensland, Brisbane, QLD, Australia

**Keywords:** COVID-19, control strategy, challenges, equity, global pandemic

## Abstract

Public health crises are challenging for governments and health systems, and coronavirus disease 2019 (COVID-19) has presented huge challenges to humans worldwide. In the context of COVID-19 in China, we explore China's control strategies and challenges. Our analysis examines seven strategies: digital technology pandemic prevention, zero-case policy, all-staff nucleic acid testing, all-staff vaccinations, the long-term quarantine system, and the official accountability system. Additionally, it considers three challenges: repeated pandemic waves, increased downward pressure on the economy and social exhaustion. We identify the causes of these challenges, including social and natural factors, and the controls put in place. We contend that China's control strategies slowed the spread of the global pandemic and that Chinese vaccines have promoted global vaccine equity.

## Introduction

The outbreak of COVID-19 has rebounded repeatedly, and it has never been effectively controlled (1). This lack of control is manifested in four main aspects (2–5). First, the COVID-19 pandemic is still in the high-level spreading stage, and the number of reported new cases is up to 500,000 a day. One of the countries experiencing a significant resurgence is India, which experienced a sharp increase in cases in April 2021 (6). There, the pandemic has continued to worsen, and the number of confirmed cases in a single day continues to exceed 300,000. The global pandemic still exists in many countries. As of November 9, 2021, the pandemic had spread to 211 countries (regions) around the world. Second, COVID-19 still has a high fatality rate (7). As of November 22, 2021, the cumulative fatality rate was 793,651 in the United States, 143,927 in the United Kingdom, 465,662 in India, 612,722 in Brazil, 133,177 in Italy, and 264,095 in Russia. Third, there is no global consensus on pandemic prevention and control strategies. There are great differences of opinion on the importance of mask wearing and the implementation of the zero-case policy, and these debates are ongoing (4–6). Fourth, the repeated global outbreaks have put great pressure on China's pandemic prevention and control measures due to imported cases, and the risk of local disease spread caused by these cases still exists (3).

It is notable that the effectiveness of one dose of vaccine was notably lower among persons with the delta variant [30.7%; 95% confidence interval (CI), 25.2–35.7] than among those with the alpha variant (48.7%; 95% CI, 45.5–51.7). The effectiveness of two doses was 93.7% (95% CI, 91.6–95.3) among persons with the alpha variant and 88.0% (95% CI, 85.3–90.1) among those with the delta variant.Additionally, 58 studies (32 studies on vaccine effectiveness and 26 studies on vaccine safety) found that a single dose of vaccine was 41% (95% CI: 28–54%) effective for preventing SARS-CoV-2 infections, 52% (31–73%) for symptomatic COVID-19, 66% (50–81%) for hospitalization, 45% (42–49%) for intensive care unit (ICU) admissions, and 53% (15–91%) for COVID-19-related death. Two doses were 85% (81–89%) effective at preventing SARS-CoV-2 infections, 97% (97–98%) for symptomatic COVID-19, 93% (89–96%) for hospitalization, 96% (93–98%) for ICU admissions, and 95% (92–98%) for COVID-19-related death. Thus, China has faced large challenges in controlling new waves of COVID-19 (8–10).

**Figure 1:**
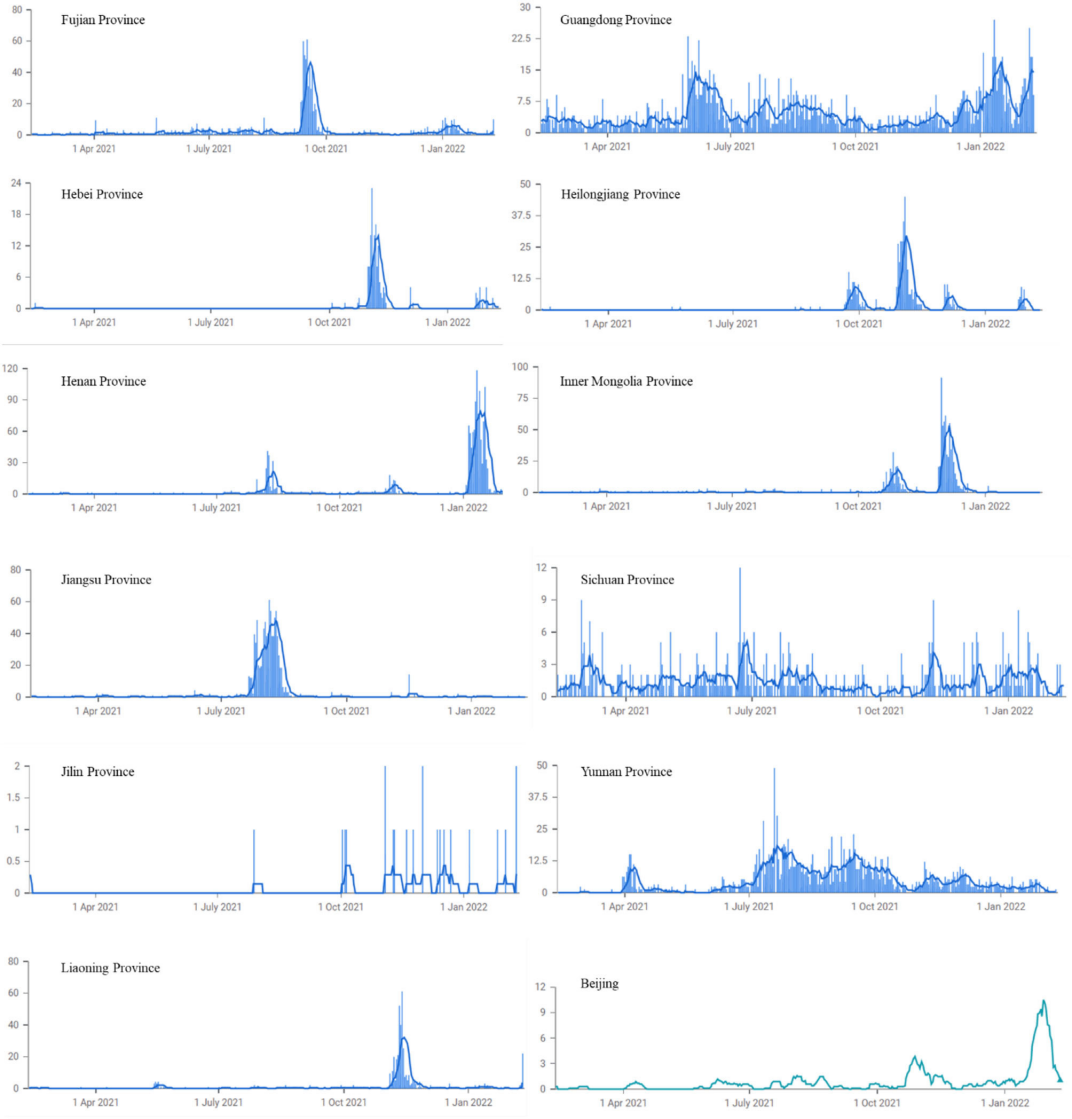
COVID-19 waves in China since 2021. Data source: Our World in Data.

With the national pandemic basically under control, sporadic cases have emerged in many parts of China, threatening Chinese people's safety and economic and social wellbeing (11–15). The repeated waves of the pandemic in China are characterized as follows: First, there are viral outbreaks at multiple locations, and the disease spreads from these epicenters (16). Then, the pandemic appears to be effectively controlled, but it occasionally recurs in a single city (region) and spreads from there to multiple cities, triggering repeated outbreaks in multiple provinces. The following provinces have experienced new waves of COVID-19: January 2021, Shijiazhuang and Hebei; February 2021, Harbin and Heilongjiang; April 2021, Ruili and Yunnan; October 2021, Alxa, Inner Mongolia, Qinghai, Gansu, Harbin, Heilongjiang, Xi'an, and Shaanxi; November 2021, Shanghai (Disney), Shandong Rizhao, Changzhou in Jiangsu, Zhengzhou in Henan, and Yinchuan in Ningxia (17–20). Second, imported cases are causing local transmission. The pandemic in China has been effectively controlled, but domestic outbreaks are being caused by infected individuals entering China from other countries and regions. Therefore, ports and airports are the most frequent sites of new cases. For example, from March to April 2021, there were occasional outbreaks in Kunming, Ruili, Yunnan and other places; and on May 20, 2021, 31 provinces (autonomous regions, municipalities) and Xinjiang Production and Construction Corps reported 24 new confirmed cases in 24 h; all were imported cases (11 in Fujian, 9 in Shanghai, 1 in Henan, 1 in Hunan, 1 in Guangdong, and 1 in Sichuan). In July 2021, Nanjing Lukou Airport was the source of an outbreak in Nanjing and Yangzhou, and in June 2020, 42 imported cases were diagnosed in Guangdong, and 35 imported cases were diagnosed in Shanghai (21). Third, COVID-19 occurs seasonally to some extent, and the symptoms are similar to those of colds, with fevers and coughs in autumn and winter, making it difficult to distinguish pandemic cases in time to put prevention and control methods in place. All of these factors cause the disease to rebound and recur (Table 1 and Figure 1) (22).

**Table 1:** COVID-19 waves in China since 2021.

**Time**	**Locations**	**Accountability people**
January 26, 2021	Harbin City, Heilongjiang Province, Daxing District, Beijing, Tonghua City, Jilin Province, Xingtai City, Shijiazhuang City, Hebei Province	19
February 14, 2021	Harbin City, Heilongjiang Province, Tonghua City, Jilin Province	402
April 21, 2021	Ruili City, Yunnan Province	1
June 12, 2021	Guangzhou City, Guangdong Province	20
July 28, 2021	Nanjing, Jiangsu Province, Dehong Dai and Jingpo Autonomous Prefecture, Yunnan Province	1
August 2 2021	Jiangsu Province, Yunnan Province, Henan Province	84
October 5, 2021	Harbin City, Heilongjiang Province, Xiamen City, Fujian Province	/
October 22, 2021	Ejina Banner, Inner Mongolia Autonomous Region	6
November 5, 2021	Changping District, Beijing	10
November 9, 2021	Ejina Banner, Alxa League, Inner Mongolia Autonomous Region, Changping District, Beijing, Heihe City, Heilongjiang Province, Shijiazhuang City, Hebei Province	10
November 14, 2021	Heihe City, Heilongjiang Province, Shijiazhuang City, Hebei Province, Chengdu City, Sichuan Province Dalian City, Liaoning Province	21

Table 1 shows the outbreak dates and locations as well as those held accountable for the outbreaks. The Chinese government has adopted a strict official accountability system, and many people across the country have been held accountable for ineffective pandemic prevention and control (23). Among them are government officials, health system officials, and officials at entry points, such as airports. The Chinese government penalizes officials, including removal, dismissal, demotion, inspection, and admonishment. The time span for accountability extends through the entire pandemic prevention and control process and involves multiple provinces, multiple levels, and multiple aspects. The accountability protocol is strong and has had a major impact (24). Therefore, China's COVID-19 prevention and control strategy and its impact on the global pandemic deserve further in-depth study.

Four sections follow this introduction. Section China's COVID-19 Control Strategies and Challenges introduces China's COVID-19 control strategies and challenges. Section The Causes of the COVID-19 Control Strategy in China states the causes of COVID-19 control strategies in China. Section Discussion and Implications: The Impact of China's COVID-19 Control Strategy on the Global Pandemic explains the impact of China's COVID-19 control strategies on the global pandemic. Section Conclusions concludes the paper.

## China's Covid-19 Control Strategies and Challenges

### Control Strategies

China's specific pandemic prevention and control strategies include digital technology pandemic prevention, zero-case policy, all-staff nucleic acid testing, all-staff vaccinations, the long-term quarantine system, and the official accountability system.

#### Digital Technology Pandemic Prevention

China's pandemic prevention and control measures make full use of new technologies such as 5G, cloud computing, artificial intelligence, and blockchain and give full play to the role of pioneering technologies in prevention and control. These tools help to determine the source of the virus and the transmission chain (25–27). In terms of digital prevention and control, China's methods include drone cameras and drone quick response (QR) code scanning services to support dynamic monitoring, tracking, and restriction of gatherings and ease the pressure on vehicles and pedestrians. These measures make full use of big data tracking and satellite communications to trace contacts, relying on health and travel codes. Moreover, they make full use of communication technology, including troubleshooting by telephone. In terms of digital technologies, during the pandemic, online apps and services promoted the orderly advancement of residents' lives with online offices, telemedicine, and shopping, prompting traditional manufacturing companies to accelerate the construction of the industrial internet. These innovations have injected new momentum into development and invigorated the economy. The pandemic has forced China to improve its social governance, make good use of technology, and accelerate the development of cities' intelligence systems (28).

#### Zero-Case Policy

This policy refers to China's strict prevention and control protocol for the COVID-19 pandemic, which prescribes active treatment to cut off the source of infection after a case is identified (1). People (groups) who have close contact with the confirmed case are medically isolated, and nucleic acid tests are used to detect and treat the disease in its early stages. These measures are aimed at eradicating COVID-19 and maintaining disease-free conditions. Some countries do not treat patients after diagnosis in hopes that the patient's immune system will be enough to heal the individual or withhold medical care until patients are very ill and have possibly infected a number of others, resulting in a worsening of the disease (10). China's zero-tolerance policy is considered excessive by some countries. Some believe that the costs of detection, quarantine, observation, nucleic acid tests, medical care and logistical support for home quarantine are unnecessary and too high (2). However, the zero-case policy of the Chinese government enabled the country to host the Beijing Winter Olympics (11).

#### All-Staff Nucleic Acid Testing

Nucleic acid testing is an important method for the prevention and control of COVID-19 infection (15). After the outbreak, localities adopted all-staff nucleic acid testing measures in urban communities and rural villages to screen for asymptomatic infections or patients with mild fever and cough symptoms (29). The Chinese government imposed specific requirements for the nucleic acid testing of all people: everyone should be checked and tested as quickly as possible, and early detection and treatment should be carried out. On September 8, 2021, the State Council issued the Guidelines for the Implementation of Nucleic Acid Testing Organizations of COVID-19 (Second Edition), which cover five dimensions: the overall requirements, organization and implementation, work preparation, work content, and relevant requirements for nucleic acid testing support teams (2–4). Time requirements are specified: “Cities with a population of more than 5 million should complete the task of nucleic acid testing for all people within 3 days and apply for national support if necessary.” The guidelines stipulate that localities should adequately prepare medical supplies for nucleic acid testing to ensure that testing is carried out in an orderly manner. With the advancement of nucleic acid testing, the Chinese government requires testing agencies to provide “24-hour service” and strive to produce results within 6 h for people who are “willing to take all the tests” to eliminate the time interval between testing and the spread of the virus (2, 5).

#### Long-Term Quarantine System

The Chinese government implemented a long-term quarantine system for people traveling overseas and to medium- and high-risk areas (22–24). Different regions have different requirements, but the 14-day medical observation quarantine is standard, and the requirements for nucleic acid testing are separate. Quarantine for medical observation is either centralized or home quarantine. With the delta virus spreading in many places, a 14 + 7, 14 + 7 + 7, or longer quarantine system has been adopted. After the outbreak at Shanghai Disneyland in November 2021, the 2 + 12 principle was required. All those who visited Disneyland and the city on the 30th and 31st also had to consciously follow the 2 + 12 principle to complete their health monitoring, which meant 2 days of closed-loop management + 12 days of strict community health management (5–8). During this period, 4 nucleic acid tests were performed on the first, second, seventh, and 14th days. So-called two-day closed-loop management meant that an individual should not go out for 2 days after the 31st. It is recommended that individuals not go to work or out of the house until two nucleic acid tests are completed (1).

#### All-Staff Vaccinations

The Chinese government strictly controls the prevention and control of the pandemic, accelerates the pace of vaccine research and development, and provides free vaccines for all people who volunteer to be vaccinated (3). The first round of voluntary free vaccination targeted qualified individuals over the age of 18. The next round targeted those aged 12–17 in July 2021, and vaccination for children aged 3–11 was carried out in November. As of November 13, 84,395 million children aged 3–11 had been vaccinated, and 49.44 million people had received booster shots. On November 5, 2021, China completed the booster immunization of 37.973 million people (1). During the vaccination process, special attention was given to susceptible areas and populations. First, vaccination efforts focused on key areas, such as port cities, border areas, large and medium-sized cities with a high risk of outbreaks and areas with clusters of previous outbreaks. Second, vaccination efforts were directed at key members of the population, including cold chain practitioners, healthcare providers, government agency personnel, employees of enterprises and institutions, college students and faculty, service personnel of large-scale supermarkets, and relevant staff members of transportation, logistics, and welfare institutions that guarantee the operation of society (30).

#### Psychological Counseling During the Pandemic

The Chinese government attaches great importance to psychological counseling to relieve the anxiety and irritability caused by the prevention and control of the pandemic, strengthen life guidance, and ensure psychological stability (2). On March 18, 2020, to further strengthen psychological counseling and psychological intervention for key populations, the State Council issued the “COVID-19 Pandemic Psychological Counselling Work Plan” (4). The plan requires all localities under its guidance to study and judge phased changes in the psychology of all people in real time, adjust the focus and response measures of social psychological services according to the time and situation, maintain the population's mental health, and promote social harmony and stability. One specific psychological counseling methods is to open a psychological counseling hotline so that residents can call and talk with a counselor about negative emotions such as panic, anxiety and depression. A second provision of the plan is online counseling on topics chosen to help manage individuals' negative emotions, coordinate family relationships, and create a positive emotional atmosphere (8, 9). A third provision is to create and disseminate motivational music in the form of songs that channel emotions and inspire confidence in individuals who feel trapped by the pandemic and to guide and encourage the music creators (12). For example, encouraging songs released in Yangzhou in July 2021 included “Love Drunk Yangzhou” and “Come on Yangzhou” (1, 2). In August 2021, “Wuyang Central Plains” was released in Henan. In October 2021, the pandemic song “I Will See You, Ejina,” played an important role in cheering up the population in Inner Mongolia.

#### Official Accountability System

The accountability system holds responsible those who violate pandemic prevention regulations, including both officials and citizens (5). China's pandemic prevention and control protocol implements a strict official accountability system. Officials who fail to perform their duties in the pandemic are held accountable, and corresponding penalties, including dismissal, demotion, suspension, warning, or filing a case for review, are imposed based on the ineffectiveness of prevention and control in their jurisdictions (Table 1) (1). This strict accountability system has ensured the continuous and effective development of pandemic prevention and control. Individual citizens are also held accountable. Those who deliberately conceal their true itineraries, refuse or evade nucleic acid testing, and spread rumors are investigated to determine their personal legal responsibility. Individual citizens suspected of violating the prevention and control of infectious diseases are also investigated. For example, in July 2021, Mrs. Mao, who came to Yangzhou from the medium-high-risk area of Nanjing Lukou Airport, was suspected of violating the pandemic prevention regulations and was investigated in accordance with the law. On November 17, 2021, the People's Court of Pingxiang City, Guangxi, held a court hearing on the case of Chinese citizen Cao for the crime of obstructing frontier health and quarantine and sentenced him to 2 years of imprisonment with a probation period of 3 years and a fine of RMB 200,000. On October 26, 2021, Anhui Lu'an reported a case involving two doctors who were sentenced to fixed-term imprisonment for 1 year and 3 months for treating patients with fever without authorization (10–14).

### Challenges Facing China's Pandemic Prevention and Control

The challenges facing China's pandemic prevention and control are manifested in three main aspects: repeated pandemic waves, increased downward pressure on the economy and social exhaustion.

#### Repeated Pandemic Waves

Repeated waves of the disease are not only the current situation in China but also a major challenge to the future prevention and control of the pandemic (31). The COVID-19 strain continues to mutate during its spread, increasing the difficulty of pandemic prevention and control. At present, the pressure of pandemic prevention and control in China comes mainly from imported cases, which have led to sporadic outbreaks in some parts of China that cannot be eliminated. Coexisting with the virus has become a way of life. In the process of spreading, the COVID-19 virus has continuously mutated, and its infectivity has continued to increase, posing new challenges to China's pandemic prevention and control. This situation requires a high degree of vigilance and continued study of new COVID-19 variants (32).

#### Increased Downward Pressure on the Economy

The downward pressure on the economy has increased, as the pandemic has had a great impact on the global economy. Although some countries have adopted monetary and fiscal stimulus policies, the extent to which the global economy can be boosted remains to be seen. The global economic downturn will also have an impact on China's economy and affect domestic economic recovery (33).

Social exhaustion: social psychology plays an important role in overcoming the pandemic.

#### Social Exhaustion

Since the outbreak of COVID-19, people have experienced a 2-year tug-of-war with the disease. In China, although the pandemic is basically under control, repeated waves have challenged people's psychological wellbeing. People's mental health has undergone changes at different stages of the pandemic (10–15). Since the pandemic has repeatedly occurred, people have become prone to fatigue, paralysis, carelessness, and even blind optimism. This creates a mentality of underestimating the disease and the part played by chance, which challenges the prevention and control of the pandemic (1–3).

## The Causes of The Covid-19 Control Strategy In China

China's pandemic prevention and control strategy has been formed under the comprehensive influence of natural and social factors. Natural causes include population characteristics and the strong infectivity and rapid spread of the virus; social factors include putting people and life first, meeting economic development needs, and considering political system characteristics.

### Population Characteristics

The population of China presents a sharp contrast between high population density in the eastern region and relatively low population density in the western region (2). The dense population distribution in the eastern region, with high mobility, close contact, and highly overlapping living circles, necessitates stronger requirements for pandemic prevention and control. It is necessary to adopt a zero-case policy and impose strict prevention and control measures to respond effectively.

The population of China is increasingly aging (5). According to the Seventh China National Census Bulletin (No. 5), the population aged 60 and older now accounts for 18.7% of the entire population, and 13.5% of this age group is over age 65. The elderly population is afflicted mostly with common diseases, weak resistance, infection vulnerability, and poor physical fitness. The mortality rate of the elderly population, once infected, is much higher than that of other age groups (1). The recovery rate of the elderly population is lower than that of other age groups, which means that COVID-19 is likely to cause severe illness and to be life-threatening. Strict prevention and control measures must be taken to effectively address this issue (7, 8).

### The Virus Spreads Quickly

COVID-19 is highly infectious, mutates during transmission, and increases in infectivity. At present, the main strain of COVID-19 is the delta strain discovered in India in October 2020 (1–4). Delta is more transmissible than earlier strains of COVID-19 before it mutates, and it continues to mutate during its transmission. In some countries, multiple mutant strains have appeared (6). On July 29, 2021, the World Health Organization (WHO) stated that 8 more countries and regions had discovered the Delta COVID-19 variant virus. As of September 22, 2021, 185 countries and regions had been infected with the Delta variant, while the virus continued to mutate (34).

### A Highly Infectious Disease

COVID-19 is highly infectious. Infection methods can be divided into two types: contact and non-contact. The contact type can spread through the air, droplets, and contact with an infected person or object (2). Contact infection is embodied in virus transmission through the use of common objects, such as elevator buttons, door handles, and delivery items. Infection through the eyes is also a type of contact infection. Non-contact infection involves coming into contact with viral particles, which can stay in the air and remain infectious for up to half an hour (4). In terms of transmission distance, COVID-19 can spread through droplets over a distance of 1–2 meters. Regarding the consequences of infection, COVID-19 can cause major damage to the lungs as well as chest tightness, dyspnea, emphysema, and palpitation. Even after recovery, patients with COVID-19 have a higher potential risk of sequelae (6).

Social factors: put people and life first, recognize the needs of economic development, and consider the characteristics of the political system.

### Put People and Life First

The Chinese government puts people at the center of all its work, and people's lives come first. Government departments, medical workers and Chinese society are doing their best to protect every life (22–25). Community workers have implemented meticulous steps to ensure residents' living needs, guaranteeing grocery items, such as grain, oil, rice, noodles, vegetables and meat, and other necessities, such as gas, water, electricity, and medicine. The government is assisting families in need of medicines by helping them with purchasing and home delivery (20). Governments at all levels have opened supermarkets and vegetable farms in an orderly manner, stabilized prices, and allocated materials to ensure that residents' daily needs are met. Industry has increased the production of medical necessities, ensured the production capacity of masks, and stabilized the supply of masks (1).

### Economic Development Needs

Economic development requires a safe and stable environment. The Chinese economy is facing a critical period of transforming the economic development mode, adjusting the economic structure, and achieving high-quality economic development (28, 29). A safe and stable environment is an important guarantee for the high-quality development of the Chinese economy. Pandemic prevention and control work has provided important support for the restoration of economic development, ensuring the human, material, and financial resources required for economic construction. In addition, pandemic prevention and control have promoted production recovery and protected lives (4, 5). Only pandemic prevention and control can ensure economic recovery and production development and fundamentally protect people's lives. Stable economic development demands require strict prevention and control strategies to control the pandemic as soon as possible (10).

### Political System Requirements

China has implemented a system of people's congresses, as the people are the masters of the country. In China's political system, the government is centered on the people; the people are supreme, and life is supreme (1, 2). Thus, ensuring the safety of people's lives is the fundamental starting point and goal of the Chinese government's pandemic prevention and control work. China's pandemic prevention and control policy is a vivid manifestation of the government's application of the concept of the people's supremacy. Leading cadres at all levels in China always remain at their posts. Grassroots cadres care about serving the masses and promptly solve their constituents' difficulties and problems (5).

Under major pandemic prevention and control conditions, people actively participate in the protocols (35). Citizens take the initiative to report their health codes and itinerary codes, report their history of travel in high-risk areas, actively cooperate with the quarantine policy, make personal contributions within their capacity to pandemic prevention and control, and take responsibility for themselves and society. The prevention and control effort in China is a major feat carried out by the Chinese government and people.

## Discussion and Implications: The Impact Of China'S Covid-19 Control Strategy On The Global Pandemic

### China's Fight Against the Pandemic Slows the Spread of the Global Pandemic

As the largest developing country in the world, China has one-quarter of the global total population. Rapid and effective pandemic control measures ensure public health safety in China and reduce the pressure on the world's pandemic prevention and control response (1–4). While the pandemic in China is under effective control, the country will promptly resume work and restore its economic production capacity. China not only guarantees the supply of its own masks, protective clothing, ventilators and other pandemic prevention supplies but also provides the world with scarce pandemic prevention materials to aid in global pandemic prevention and control (13).

China has shared its experience in fighting the pandemic with the world and sent experts to assist other countries in controlling the pandemic (34–36). The Chinese government has unreservedly shared its valuable experience through various means, such as videos and internet content, to provide other countries with experience in pandemic prevention, control, treatment and care. China's sharing has been appreciated by Serbia, Iran, Japan and other countries. Serbian President Vucic, Iran's minister of health, and the Komeito Party of Japan expressed their gratitude and appreciation for China's sharing of experience in a variety of ways (15, 16).

China's successful fight against the pandemic has inspired the world's confidence in its ability to defeat the pandemic. Confidence in victory over the pandemic is the spiritual force needed to overcome it (2). The successful practice of China's pandemic prevention and control efforts has provided an actionable plan for global pandemic prevention and control. China has found a scientific basis for fighting the pandemic, inspired the world's determination to overcome panic, and strengthened confidence in victory (35).

### China Promotes Global Vaccine Equity

The speed of vaccine research and development varies from country to country, and there has been inequity in global vaccine distribution (1). As a scarce resource, vaccines have become a new tool for some countries to artificially set additional conditions and impose hegemony. The research, development, production and provision of vaccines in China can ensure more fairness and justice in countries lacking vaccines and help them obtain a safer and more sanitary environment (3).

#### Current Status of Global Vaccines

At present, the development of vaccines is dominated by China, the United States, the United Kingdom, Germany and other countries. This has become an important aspect of competition among countries. However, it is difficult for developing countries to provide sufficient funds and scientific and technological support to develop vaccines. With the repeated waves of the pandemic, countries supporting research and development have accelerated vaccine research and clinical trials. However, the gap between China, the United States, the United Kingdom, and Germany and developing countries continues to widen (5–7).

Few vaccines are available in developing countries. The lack of vaccine development capabilities in developing countries, coupled with the inherent weakness of their medical systems, makes it difficult for them to fight COVID-19, resulting in serious consequences (9). It is difficult for developing countries to obtain vaccines from high-income countries, resulting in unfair vaccine distribution, inequitable vaccination numbers, and even more serious global inequities. In terms of vaccine distribution, the North-South divide is prominent. At present, the number of available vaccines is seriously unbalanced between rich developed countries and developing countries. The former may even hoard more vaccines than they need, while developing countries cannot obtain vaccines at all. There are also distribution problems within developed countries. In some countries, the problem of inequality between the rich and the poor is prominent, and it is easier for the rich to obtain vaccines (35–37).

Developed countries have a relatively sufficient number of vaccines, and vaccination is easier to obtain, especially for wealthy people in developed countries, where the vaccination rate is higher. For developing countries, because it is difficult to obtain vaccines, it is difficult to increase the vaccination rate and to popularize vaccines (38, 39).

Regarding global vaccine hegemony, the role of the vaccine itself is limited to carrying the moral responsibility of maintaining life safety (2). However, some rich countries use vaccines as a bargaining chip to compel other countries to agree to extraneous demands. When developing countries purchase vaccines, additional mandatory terms are added to the negotiation, which increases the burden on poor countries (5, 6). For example, in return for vaccines, the US pharmaceutical giant Pfizer required Argentina to mortgage bank reserves, military bases and embassy buildings; required Brazil to use overseas assets as guarantees for the purchase of vaccines; and agreed with the signatory countries that when disputes arise, they will be resolved through private arbitration in accordance with New York law (37).

In short, the global distribution of vaccines is unbalanced and unfair between North and South, and the gap in the safety environment caused by vaccines has widened, resulting in imbalances in social and environmental security.

#### China's Vaccine Promotes Vaccine Fairness

Many vaccines developed in China have been urgently approved for use in many countries, and several have been authorized by the WHO for emergency use (1). The Sinopharm and CoronaVac vaccines were included in the emergency-use list released by the WHO. In October 2020, China joined the “COVID-19 Vaccine Implementation Plan.” Envoys of various countries and representatives of international organizations believe that China is taking concrete actions to promote the equitable distribution of vaccines and fulfill its commitment to make the COVID-19 vaccine a global public product (2–5). On November 8, 2021, the British government announced that it would recognize the WHO's November 22 emergency-use list of COVID-19 vaccines, including Sinopharm and CoronaVac. Travelers who have been fully vaccinated with the vaccines on the list do not need to be quarantined when they arrive in the UK (26).

China donates vaccines to underdeveloped countries. China regards vaccines as a global public product and believes that everyone should have access to them, especially citizens of developing countries that are in urgent need (38–40). According to relevant media reports, countries including Chile, Brazil, and Colombia have urgently approved the use of Chinese vaccines, and Uruguay, Mexico, Peru and other countries will receive these vaccines in the near future. China has made it clear that it will prioritize developing countries (41). At the same time, due to China's strong vaccine production and manufacturing capabilities, it is capable of providing vaccines to many countries (42).

### Can the Chinese Model Be Copied to Other Countries?

China has adopted strict prevention and control policies to slow the spread of COVID-19. This approach truly saves lives and achieves economic development, but at a huge cost (43–45). We do not yet know whether is a low-cost strategy or a high-cost strategy from the long-term view compared to the strategies of other countries (Table 2). We question whether the Chinese model can be copied to other countries. First, there is no doubt that the China model has made a great contribution to slowing worldwide pandemic development, as China has provided COVID-19 vaccinees to many countries. Second, it is possible that other countries can apply selected COVID-19 control strategies from China, such as digital technology applications to track the fast development of new cases, as there is no one-size-fits-all approach. In December 2021, the new coronavirus variant, omicron, was first identified in South Africa and has been spreading quickly around the world. It has an exceptionally high number of mutations, and those mutations appear to make it more transmissible than the delta variant. This highly contagious COVID-19 variant is causing an increase in cases and a high demand for testing. Digital technology has played a huge role in China in detecting new cases and stopping the spread.

**Table 2:** COVID-19 prevention and control status in different countries and regions.

**Country or region**	**Prevention and control measures**	**Prevention and control effect**	**Prevention and control trend**
China	Digital technology pandemic prevention, zero-case policy, all-staff nucleic acid testing, all-staff vaccinations, long-term quarantine system, and official accountability system	To achieve dynamic clearing, in 2021, the economy will grow by 8.1% over the previous year	Normalized prevention and control
EU	Restrictions on gatherings in 2021, requiring the use of vaccine passes, and recommending masks during rush hour on public transport	Cases remain high.	From strict to relaxation, unlocking and allowing travel without isolation and testing
United States	Not enough attention was paid early. Although prevention and control measures have been taken, such as “maintaining social distance,” a travel ban has been imposed on Europe, and US nationals have been advised to reduce unnecessary travel. Several states issued stay-at-home orders. However, the states did not implement these effectively, and they have disagreed and quarreled about the implementation of prevention and control measures.	The United States has become the country worst hit by the new crown pneumonia pandemic. Nearly one-fifth of the world's known confirmed cases are in the United States.	From February 2, 2022, the United States will no longer count the number of new cases and deaths and announced the cancellation of the “mask mandate”
Japan	Catering restaurants are required to shorten their business hours, the public is required to reduce unnecessary outings, and the public is called on to continue to take good care of personal protection, wear masks and disinfect frequently, and avoid crowded areas. In addition, Japanese people have the cultural habit of wearing masks, which is effective for pandemic prevention and control. Vaccination rates are high.	The control effect is better.	Ease pandemic control. The number of confirmed cases increases.
Singapore	Distribute self-checking devices to enterprises, reduce non-essential social activities, and implement home notice and social distancing measures in the form of legislation. Violators will be fined and imprisoned. The vaccination rate is 90%.	The control effect is better.	Gradually ease pandemic prevention and control measures. In February 2022, the number of children infected by omicron increased. The omicron variant is currently causing a new round of outbreaks in Singapore.
Russia	Strict prevention and control measures, strictly guard the border to prevent the import of overseas pandemics, wear masks and protective gloves in public, maintain social distance, try to avoid participating in gathering activities, start all-Russian paid vacations, and reduce the flow of people.	The situation of the new crown pandemic in Russia is still very serious.	Keep the strict measures
South Africa	A five-level “lock order” with different prevention and control requirements is formulated, and people's degree of social contact is controlled according to the pandemic situation to reduce personnel contact. People are encouraged to be vaccinated.	It is still the country most severely affected by COVID-19 on the African continent.	The willingness to be vaccinated against COVID-19 is low.
India	Promoting COVID-19 testing and vaccinations, imposing curfew at night, reducing store hours, and teaching online	The number of infections has climbed several times, resulting in an explosive increase.	In February 2022, the pandemic situation is tight, and many places in India have comprehensively upgraded prevention and control measures.

*Only several cases are listed, and the detailed information is not exhaustive*.

It is worth noting that scientists reported that in the population in the South African study, the Pfizer vaccine's effectiveness against infection dropped to ~30% for the omicron variant, compared with ~80% against the variant that preceded omicron. The researchers found that two shots of the Pfizer vaccine still offered ~70% protection against hospital admission for COVID-19 in the population examined in the study. That percentage represented a drop from 90% observed during the previous surge in South Africa, when the delta variant dominated. These changes have alerted us that the spread of COVID-19 cannot be stopped only by vaccines. Moreover, the United States, Australia, the UK and some other countries have experienced rising numbers of new omicron cases and more related deaths. Therefore, we doubt ‘living with the virus' is a better choice.

## Conclusions

The important takeaway from China's experience in pandemic prevention and control is to respect science. Chinese scientists analyzed COVID-19 calmly and scientifically. The researchers paid close attention to the situation, studied the mutations, analyzed the pandemic with a scientific attitude, and prevented and controlled the spread with scientific methods. The spirit of science predicts the trend of the pandemic and indicates a need for coordinated prevention and control measures. Pandemic prevention and control require the unity and cooperation of human society and every country. The prevention and control of the pandemic in China reflect interaction, assistance, and mutual benefit at all levels, from central to local. Global pandemic prevention and control require systematic and comprehensive prevention and control.

## Author Contributions

DD and RZ: conceptualization, writing—original draft, and writing—review and editing. DD: funding acquisition and resources. RZ: project administration and supervision. Both authors have read and agreed to the published version of the manuscript.

## Conflict of Interest

The authors declare that the research was conducted in the absence of any commercial or financial relationships that could be construed as a potential conflict of interest.

## Publisher's Note

All claims expressed in this article are solely those of the authors and do not necessarily represent those of their affiliated organizations, or those of the publisher, the editors and the reviewers. Any product that may be evaluated in this article, or claim that may be made by its manufacturer, is not guaranteed or endorsed by the publisher.
